# Obsessive-compulsive symptoms in patient with schizophrenia: The influence of disorganized symptoms, duration of schizophrenia, and drug resistance

**DOI:** 10.3389/fpsyt.2023.1120974

**Published:** 2023-02-27

**Authors:** Georgi Panov, Presyana Panova

**Affiliations:** ^1^Psychiatric Clinic, University Hospital for Active Treatment “Prof. Dr. Stoyan Kirkovich”, Trakia University, Stara Zagora, Bulgaria; ^2^Department of Psychiatry and Psychology, University “Prof. Dr. Asen Zlatarov” Medical Faculty, Burgas, Bulgaria; ^3^Medical Faculty, Trakia University, Stara Zagora, Bulgaria

**Keywords:** resistant schizophrenia, obsessive–compulsive symptoms, consensus, disorganized symptoms, dimensional obsessive-compulsive symptoms scale, schizophrenia, positive and negative syndrome scale, duration

## Abstract

**Background:**

Schizophrenia is a chronic mental disorder with a many-faced clinical presentation. Obsessive–compulsive symptoms are often part of it. The characteristics of the clinical picture and the course of schizophrenia are factors related to both the resistance and the manifestation of obsessive–compulsive symptoms. Our study aims to establish the relationship between the peculiarities of the schizophrenia process and the influence of resistance on the expression of obsessive–compulsive symptoms.

**Methods:**

A study was conducted on 105 patients with schizophrenia. Of them, 39 are men and 66 are women. The evaluation of the effectiveness of the treatment showed that 45 were resistant to the applied therapy, while the remaining 60 responded. Clinical assessment of patients was performed using the Positive and Negative Syndrome Scale (PANSS) and Brief Psychiatric Rating Scale (BPRS). Assessment of obsessive–compulsive symptoms (OCS) was conducted with the Dimensional obsessive–compulsive symptoms scale (DOCS).

**Results:**

In 34% of all patients, we found clinically expressed obsessive–compulsive symptoms. In 40% of the patients with resistance, we found clinically expressed obsessive–compulsive symptoms, which are within the range of moderately expressed. In 30% of the patients in clinical remission, we found obsessive–compulsive symptoms, but mildly expressed. We found a statistically significant relationship between the severity of OCS and the disorganized symptoms and the duration of the schizophrenia process. No differences were found in the expression of OCS in patients of both sexes.

**Conclusion:**

We registered both an increased frequency and an increased expression of obsessive–compulsive symptoms in patients with resistant schizophrenia. These symptoms were positively associated with disorganized symptoms and duration of schizophrenia. No relationship was established with the positive, negative symptoms, as well as with the gender distribution.

## 1. Introduction

Schizophrenia is a mental disorder of unknown etiologies and complex clinical presentation. The disorder has many intertwined symptoms outside of the typically psychotic ones—delusions and hallucinations (1). In addition to mental symptoms, numerous other concomitant changes have been reported, such as impaired brain connectivity, changes in metabolism, the opioid system, and immunological parameters (2–6). All these data give us reason to think that schizophrenia is a systemic process in which mental symptoms as clinical presentation are part of the overall picture of the disorder.

When schizophrenia is discussed in the foreground, we are used to thinking about the dopamine hypothesis, which is the basis of the therapeutic models used in practice (7, 8). On the other hand, it does not give us the opportunity to explain many of the symptoms as well as the resistance in a large part of the patients. Understanding resistance in patients with schizophrenia requires a complex approach of dissection (9–11) of the underlying chemical, biochemical, metabolic, neurotransmitter, neuronal migration, neuronal connections as well as the assessment of the involvement of the different parts of the brain. Systemic disorders in tryptophan-kynurenine metabolism have been found to be the basis of many mental disorders, including schizophrenia (12). In schizophrenia spectrum disorders, inverse relationships have been found between tryptophan levels and the glutamate neurotransmission, and these complex metabolic breaches largely underlie the lack of integrity in the white matter of the brain in patients with schizophrenia (13).

Data highlighting impaired connectivity in patients with schizophrenia show that some brain regions, such as the ventromedial prefrontal cortex, associated with the modulation of fearful experiences (14) have reduced connectivity with other brain regions. An anti-correlation was found between the ventromedial prefrontal cortex with the dorsolateral prefrontal cortex and the supplementary motor cortex in patients with schizophrenia. These relations have been found to be associated with high scores using the PANSS scale (15). These data support the opinion of some authors that schizophrenia can be considered as a disconnection syndrome (16). In addition, functional interrelationships and breaches between the central and autonomic nervous system mediated by the ventromedial prefrontal cortex have been found to be related to heart rate regulation and thus associated with increased cardiovascular risk in patients with schizophrenia. This risk is increased both by the schizophrenic process itself and by the influence of the antipsychotic therapy used (17–19).

Frontal lobe dysfunction has been found in both schizophrenia and obsessive–compulsive disorder (20, 21). Research shows that in these diseases, there is an involvement of two partially independent neuroanatomical systems: the ventromedial prefrontal cortex and the dorsolateral prefrontal cortex (21). On the other hand, there are also data that in obsessive–compulsive disorder, as mentioned above, there is also a loss of connectivity, disconnection syndrome, in separate brain regions, mainly between the frontal lobe and parts of the limbic system (22). Corresponding to these data are the registrations and evaluation of alpha oscillations using neurophysiological methods (EEG). They additionally show disturbances in the connectivity in the DMN (default mode network) reflecting in the clinical symptoms in patients with schizophrenia and obsessive–compulsive disorder (23, 24).

The clinical relationship between schizophrenia (SZ) and obsessive–compulsive disorder (OCD) was reported more than a century ago with the work of Westphal in 1878, who provided the first description of obsessive–compulsive disorder (OCD) among (SZ) patients. Later, this connection was emphasized by other authors: Janet in 1903 and Bleuler in 1911 (25–28). In 2011, a meta-analysis of 50 studies reported that the prevalence of all anxiety disorders among patients with schizophrenia was about 38.3% (29). The prevalence of OCD is estimated at 12.1%, substantially higher than in the general population, incidence around 1.9–2.5% (30). These data were confirmed in a meta-analysis in 2013 on 3,978 patients suffering from (SZ), in which the presence of OCD was 12.3% and obsessive–compulsive symptoms in 30.3% of patients (31). The same authors also found a relationship with the longer duration of the schizophrenic process in patients with comorbid OCD (31). These results are in accordance with previous studies estimating the prevalence of OCD in schizophrenia at around 12%, others between 9.1 and 10.6%, while prevalence of obsessive–compulsive symptoms is at 30% (29, 32, 33). The analysis of 172 patients with schizophrenia, schizophreniform disorder, or schizoaffective disorder showed a high percentage of patients with comorbid OCS—48.9% (34). The analysis focused on the presence of OCS among patients with SZ gave rise to the concept of “schizo-obsessive disorder” as a specific clinical entity (35–37). A study indicates that the average time to onset of symptoms of SZ in OCD patients is 2.5 years and shows that first-degree relatives of OCD patients are also at increased risk of developing schizophrenia (38). Similar results have been reported in other investigations (39, 40).

The influence of obsessive–compulsive symptoms on the course of schizophrenia has been analyzed in other studies. A study of patients with a first psychotic episode shows that the presence of OCS in patients with schizophrenia is associated with a greater severity of the global assessment of both positive and negative symptoms. This severity was also reflected in the doses of antipsychotic medications used—much higher in the group of schizophrenic and comorbid OCS (41). Other analyses show opposite results. They found no correlation between obsessive compulsive symptoms and the presence of positive and negative symptoms in patients with schizophrenia (42).

According to other authors, OCS are not related to the points on the PANSS scale, the expression of psychotic symptoms, but independently reduce social functioning. In this analysis, the authors analyzed patients with pronounced social dysfunction assessed by SOFAS scale – below 60 points (43). Research by some authors gives them reason to believe that the presence of OCD is even protective against the development of psychotic symptoms (44). Others registered that social functioning depends not directly on disorganized but on the interdependence between disorganized and obsessive–compulsive symptoms (43).

When assessing the presence of OC symptoms and OCD in patients with schizophrenia and following the course and prognosis for a 5-year observation period, authors found no differences in the course of schizophrenia in patients with OC symptoms and OCD, respectively, and those without the presence of accompanying ones. They found in the majority of them, about 70%, mainly a fluctuating course (34).

Authors found an association between OCS and the use of antipsychotic medications (45, 46). Another analysis, which to a certain extent can be contrasted with the previous study, did not find a relationship with the use of antipsychotic medications and obsessive–compulsive symptoms except in patients treated with clozapine and after a period of 6 months (47). Other observations showed that often in the course of treatment, OC symptoms appear as part of the symptomatology associated with the use of second-generation antipsychotics. They recorded the appearance and persistence of OC symptoms in the group of patients treated with clozapine and olanzapine in contrast to the low comorbidity of those with amisulpride, AMS, and aripiprazole, ARZ (48, 49). On the other hand, the same medications (risperidone, olanzapine, and even clozapine) are also used in the treatment of OCD patients as part of drug therapy (50–52). A meta-analysis of the effects of antipsychotic medications in OCD patients by other groups of authors showed that they were used as adjunctive therapy to antidepressants to enhance treatment effects, and nearly one in three non-responders to antidepressants alone improved after addition of antipsychotic medication (53, 54). On the other hand, it should be noted that, to a large extent, most likely the relationship between antipsychotic medications and the occurrence of OCD in patients with schizophrenia is clearly overstated. A large-scale analysis in Sweden showed that in patients with schizophrenia (*n* = 58,336) and OCD (*n* = 19,814), patients with schizophrenia were seven times more likely to be later diagnosed with OCD compared to the general population. An analysis of the influence of antipsychotic medication on this relationship showed that the association was not significant (38). A research conducted to compare the influence of the relationship between pharmacological factors, therapy, and genetic predisposition shows that the main link between schizophrenia and obsessive–compulsive disorder is genetic predisposition (48).

In patients with schizophrenia, as well as in those with other chronic diseases, it has been found that a significant part of them are resistant to the ongoing treatment. Various attempts have been made to define resistant patients (55–58) and due to the differences between them, it is consensus opinion formed. The proposed consensus rating model for resistant schizophrenia (TRS) defines it as persistence of symptoms despite ≥2 trials of antipsychotic medication of adequate dose and duration. TRS occurs in up to 34% of patients with schizophrenia (59).

From the presented data, we found that patients with schizophrenia have high comorbidity with obsessive–compulsive symptoms. However, they do not provide sufficient information about the difference in the prevalence of obsessive–compulsive symptoms in patients with resistance and in those in clinical remission as well as the relationship with the clinical peculiarities of the course of schizophrenia in them.

These findings provide the basis for the stated purpose of our study: to determine the differences in the prevalence and expression of obsessive–compulsive symptoms in treatment-resistant patients and to establish the relationship with the peculiarities of the course and clinical characteristics of schizophrenia.

## 2. Materials and methods

### 2.1. Participants

A total of 105 patients with schizophrenia with recurrent psychotic episode were observed. Patients were admitted for treatment in the psychiatric clinic after assessment of mental status in outpatient care. The consecutive appearance of psychotic episode was associated with the relevant change in behavior necessitated consultation in an outpatient setting. A structured clinical interview was used to assess the condition, according to the DSM 5 diagnostic toolkit (60). Patients assessed as having another psychotic episode were admitted for treatment in a psychiatric clinic of the University Hospital “Prof. Dr. Stoyan Kirkovich” in the city of Stara Zagora.

Upon admission, the condition was reassessed and diagnostic tests related to the accepted inclusion and exclusion criteria were conducted. Treatment with the use of antipsychotic medications was carried out according to the psychotic profile and the anamnesis data of the patients after analyzing the information on the presence or absence of effectiveness in carrying out previous medication regimens. The choice of medication therapy is tailored both to the individual characteristics of the patient and to the profile of side effects of individual medications. The goal is to achieve an effect from the application of antipsychotic therapy. A combination of different antipsychotic medications was used in a part of the patients, observing the principles for assessing resistance in patients with schizophrenia (59). In the absence of effectiveness of certain drug strategies, changes in therapy were made according to the resistance criteria (59).

There are conflicting data on the influence of individual antipsychotic medications on the possible occurrence of OCS. In connection with the search for a maximum therapeutic response from the implementation of antipsychotic therapy, which is related to the need to use different medications, combined therapy as well as changing medications in the search for effectiveness, we excluded antipsychotic medications as criteria for analysis. Another reason for this is that we cannot analyze a particular medication and its effect on OCS due to the fact that it will either be in combination or, in the absence of effectiveness, a change to another antipsychotic will have to be made. A third reason is the number of analyzed patients, who, when taking various antipsychotic medications, often in combination, could not provide reliable statistical data, even more so, that the goal of the study is to achieve the effectiveness of the treatment, and not the analysis of an individual medication with its effects on a given period of time.

The patients were observed for the period from 2017 to 2022. The observation of the patients was done in the beginning in inpatient unit, and later mainly in the patients with an effect of the treatment in outpatient conditions.

The observation of the patients and the change in their condition showed that 45 had resistant schizophrenia, while the remaining 60 achieved clinical remission.

Including criteria:

1. Diagnosis of schizophrenia according to the Diagnostic and Statistical Manual of Mental Disorders, Fifth Edition (60, 61);
2. Between 18 and 60 years of age;
3. At least primary education;

Including criteria for patients with resistant schizophrenia are those who have met the resistance criteria of the published consensus on resistant schizophrenia (59). These are:

1. Assessment of symptoms with the PANSS and BPRS scale (62, 63).
2. Prospective monitoring for a period of at least 12 weeks.
3. Administration of at least two antipsychotic medication trials at a dose corresponding to or greater than 600 mg chlorpromazine equivalents.
4. Reduction of symptoms when assessed with the PANSS and BPRS scale by less than 20% for the observed period of time.
5. The assessment of social dysfunction using the SOFAS scale is below 60.

Including criteria for patients with schizophrenia in clinical remission are those who have met the criteria of the published consensus on remission in schizophrenia (64).

The exclusion criteria are:

1. Mental retardation
2. Psychoactive substance abuse
3. Presence of organic brain damage
4. Concomitant progressive neurological or severe somatic diseases
5. Expressed personality change (According to the diagnostic toolkit of DSM 5 and ICD 10)
6. Score of MMSI below 25 points
7. Pregnancy and breastfeeding.

### 2.2. Methods

Obsessive–compulsive symptoms were assessed with the DOCS Scale (DOCS—Dimensional Obsessive–Compulsive Scale) (65).

A DOCS total score of 18 optimally distinguishes between someone with OCD and someone without a psychiatric diagnosis, while a score of 21 optimally distinguishes between someone with OCD and someone with an anxiety disorder (65).

We used the SPSS statistical package (version 26). Correlation analysis was used to investigate the relationship between obsessive–compulsive symptoms and clinical features (clinical scales—PANSS and BPRS) in patients with schizophrenia. Gender stratification was performed. A non-parametric statistical method was also used, Mann–Whitney *U* test (66).

The multiple regression analyses were used to evaluate the effect of the independent variables: PANSS positive, disorganized and negative symptoms, age, gender, age at onset, illness duration, level of education, BMI, and height (independent variables) on OCS (dependent variable).

The same group of patients were also analyzed with regard to other clinical indicators such as depressive complaints, dissociative symptoms, lateralization of brain processes, the effect of the administration of the first antipsychotic medication, and the gender-associated role in patients with schizophrenia (67–73).

All procedures were carried out in accordance with the Declaration of Helsinki. All patients received written informed consent before admission to the clinical settings and performing diagnostic tests and therapy.

## 3. Results

Of 105 patients, 45 have resistant schizophrenia and the remaining 60 are in clinical remission. The gender breakdown showed that 66 were women and 39 were men. Distribution of patients’ age, age of onset, duration of schizophrenia, BMI, height, education, and handedness is presented in Table 1.

**Table 1:** Distribution of patients’ age, age of onset of schizophrenia, duration of schizophrenia, BMI, height, education, and handedness in both groups of patients.

	Resistant SZ	Clinical remission
Age (years)	36.98	37.25
Age of onset of SZ (years)	23.04	27.37
Duration of SZ (years)	14.31	9.87
BMI	26.6022	27.2217
Height	170.11	167.38
Education (years)	11.33	11.60
Handedness (right/left)	42/3	56/4
Sex (M/F)	20/25	19/41

We find statistically significant differences between the two groups of patients in the parameters indicated in Table 1 only in relation to the age of onset of the schizophrenia process (*p* = 0.007; *t* = −2.768) and the duration of schizophrenia (*p* < 0.025; *t* = 2.278) in them.

The assessment of obsessive–compulsive symptoms with the DOCS scale in all observed patients showed a mean value of 18.02, SD 10.087, and the minimum and maximum values were 2 and 46, respectively. Of all the patients, in 36, we found on the scale more than 18 points. In the rest of the patients, the points are below this norm, where the authors of the scale indicate that no clinical OC symptoms are observed. These patients make up 34.2% of all patients.

The analysis of the relationship between the distribution of the expressiveness of the obsessive–compulsive symptomatology in both sexes showed that in men, the average value of the OCS was 18.92, the standard deviation was 1.494. For women, the mean value of OCS was 17.48, the standard error was 1.298 (*p* > 0.05).

**Figure 1:**
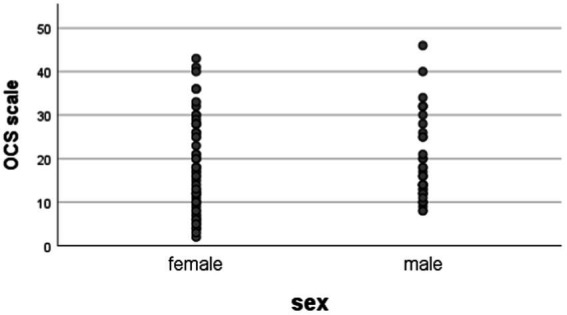
Gender distribution of obsessive–compulsive symptoms (*p* > 0.05).

The statistical analysis showed no statistical dependence between the two sexes regarding the expression of OCS (Figure 1).

In the case of females – 66, we found values above 18 according to DOCS in 24 (36.4%). Among the 39 males, we found 12 (30.8%) values on the scale above 18 points (*p* > 0.05).

In the group of patients with resistant schizophrenia, 18 patients (40%) with points on the DOCS scale above 18 were found, while in patients in clinical remission, the same number of patients were found −18 (30%) of all patients. The results show that a higher percentage of patients with clinically pronounced obsessive–compulsive symptoms was registered in the group with resistant schizophrenia.

**Figure 2:**
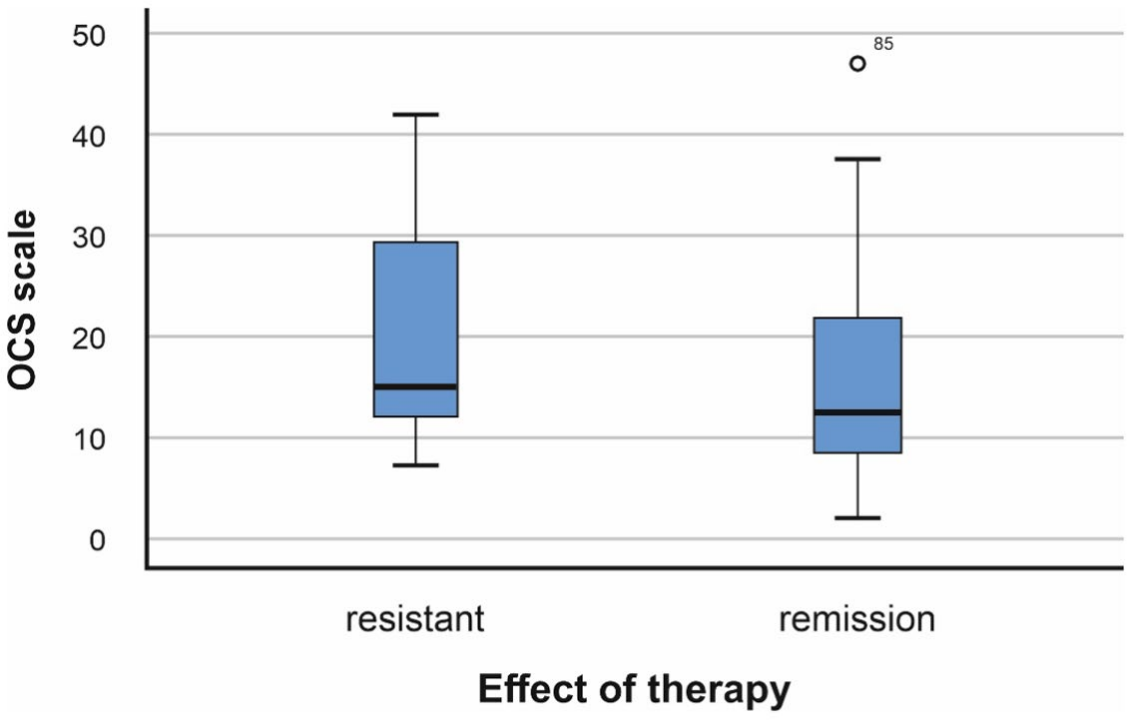
Relationship between resistance to therapy and obsessive–compulsive symptoms (*p* = 0.015, *F* = 0.648; Mann–Whitney *U* test −966,500, *p* = 0.013).

The average value on the OCD scale in the group of patients with resistant schizophrenia (RS) is 20.76, the standard deviation is 10.232. In patients in clinical remission (CR), it was 15.97 and the standard deviation was 9.554. Conducting a statistical analysis of the relationship between the expressiveness of obsessive–compulsive symptoms and resistance to therapy showed the following dependencies: Figure 2.

The conducted analysis showed the existence of a statistical relationship between resistance to treatment and the presence of higher values of obsessive–compulsive symptoms (*p* < 0.05).

Analysis of the relationship between OC symptoms and points on the PANSS and BPRS scales showed statistical dependence. It is presented in Table 2.

**Table 2:** Relationship between OC symptoms assessed by DOCS and the PANSS and BPRS scales.

	OCD scale	PANS total	BPRS
OCD scale			
Pearson correlation	1	0.230*	0.238*
Sig. (2-tailed)		0.018	0.015
PANS total			
Pearson correlation	0.230*	1	0.911**
Sig. (2-tailed)	0.018		0.000
BPRS			
Pearson correlation	0.238*	0.911**	1
Sig. (2-tailed)	0.015	0.000	

This table shows that there is high correlation between the severity of symptoms on the PANSS and BPRS scales and the level of OCS established with the DOCS Scale. In both scales, there is a statistical dependence with the presence of OCS (*p < 0.05).

Conducting a correlation analysis for the relationship with the individual sub-scales: positive, negative and disorganized, we obtained the following results presented in Table 3.

**Table 3:** Relationship between OCS assessed by DOCS and PANSS subscales.

	PANS negative	PANS positive	PANS disorganized	OCD scale
PANS negative				
Pearson correlation	1	0.493**	0.730**	0.203*
Sig. (2-tailed)		0.000	0.000	0.038
PANS positive				
Pearson correlation	0.493**	1	0.738**	0.171
Sig. (2-tailed)	0.000		0.000	0.081
PANS disorganized				
Pearson correlation	0.730**	0.738**	1	0.253**
Sig. (2-tailed)	0.000	0.000		0.009
OCD scale				
Pearson correlation	0.203*	0.171	0.253**	1
Sig. (2-tailed)	0.038	0.081	0.009	

The analysis performed shows that the highest statistical dependence is observed between the presence of OCS and the disorganized subscale of the PANSS. There is a weaker statistical relationship between OCS and the negative subscale of the PANSS. The data showed no association between OCS symptoms and PANSS-positive symptoms.

**Figure 3:**
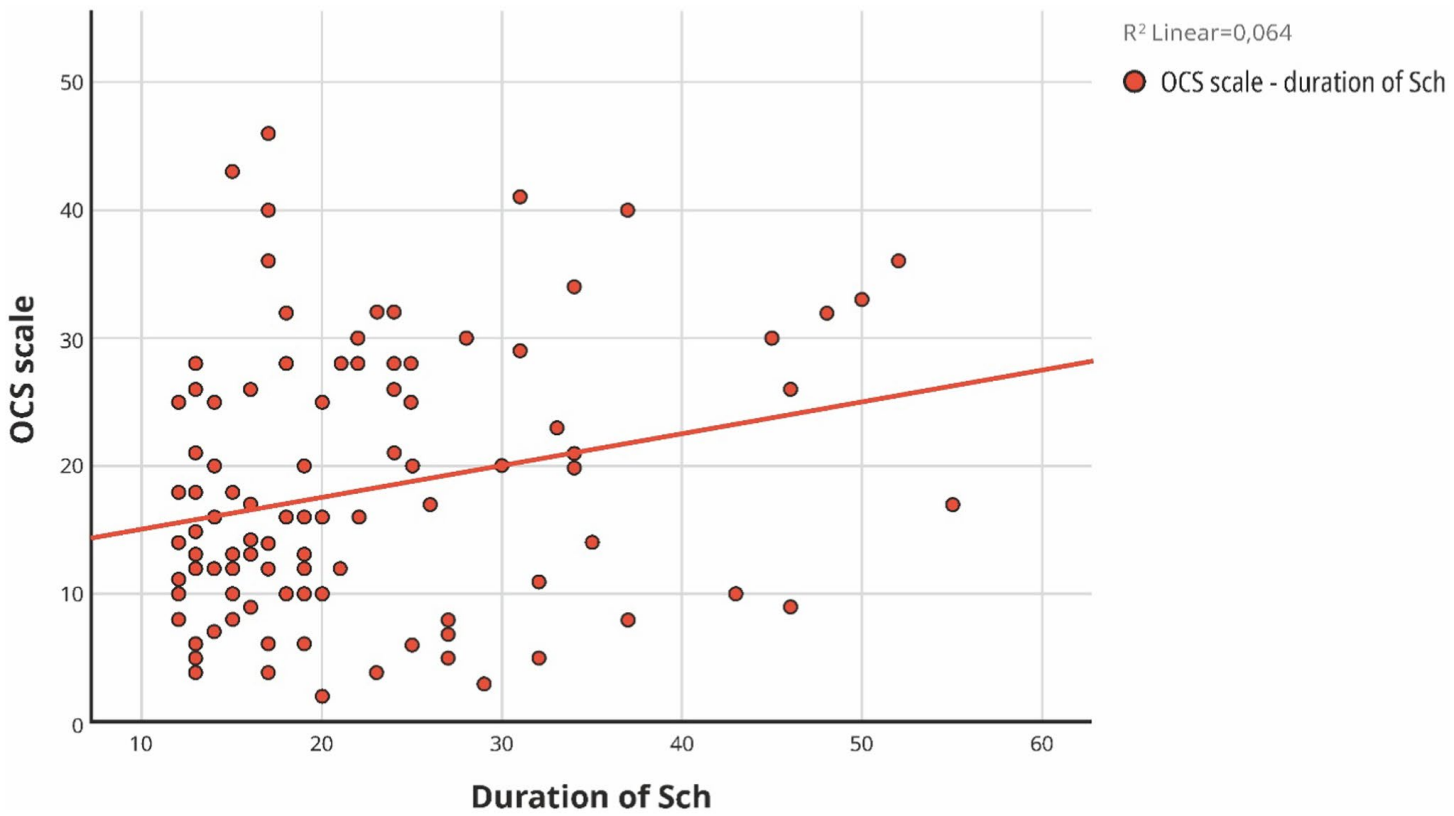
Simple Scatter with Fit Line of scale of OCS by duration of Schizophrenia.

**Figure 4:**
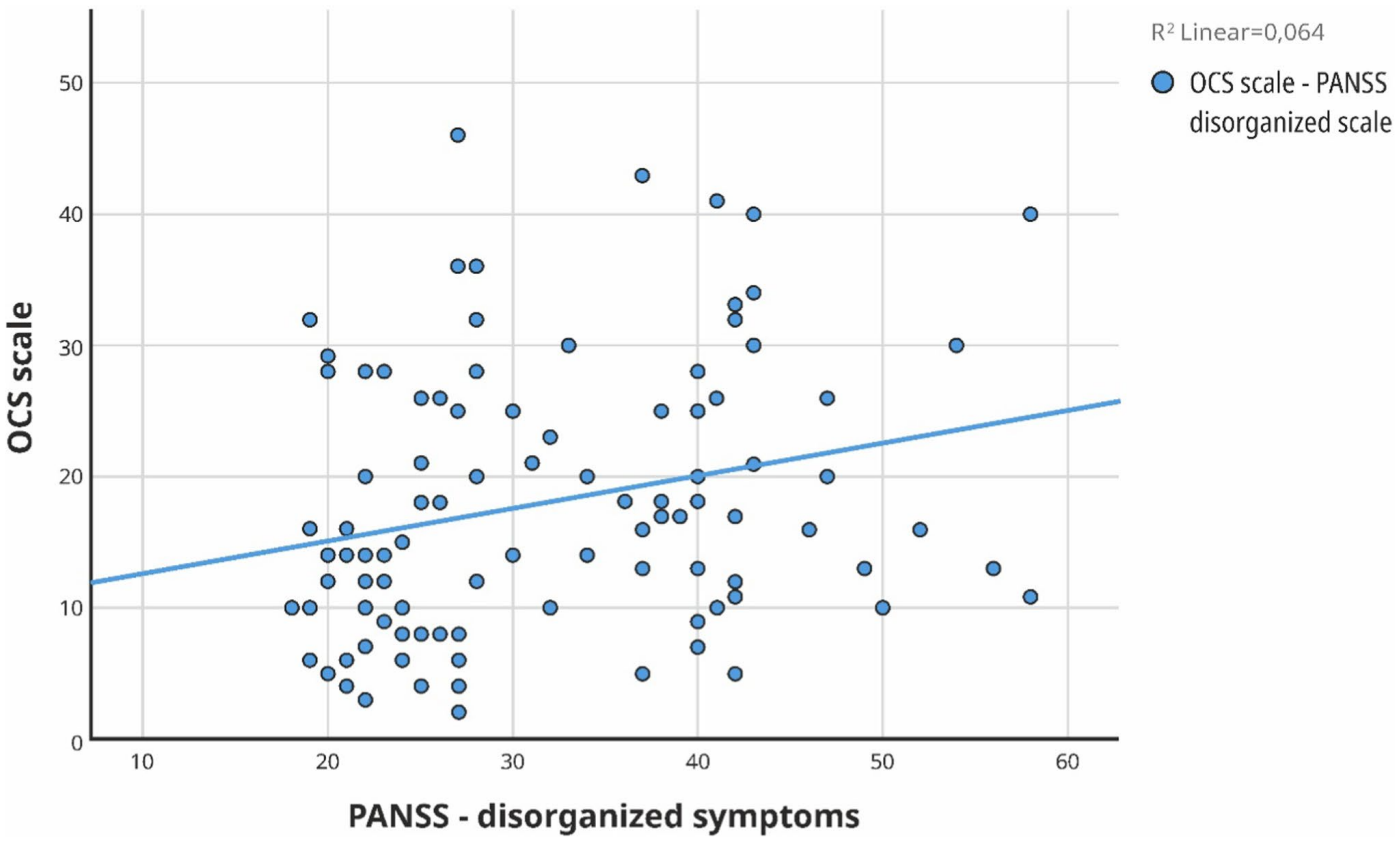
Simple Scatter with Fit Line of scale of OCS by PANSS- disorganized symptoms scale.

In order to comprehensively analyze the influence of additional factors such as the PANSS scales, body mass index, education, the onset of the disease, its duration, the age of the patients, and education on OCS, we conducted a multiple regression analysis (Table 4, Figures 3, 4).

**Table 4:** Relationship between OCS with the onset of schizophrenia and its duration.

	*R*^2^	*β*	*t*	*p* (sig)
Step 1 PANSS disorganized	0.064	0.253	2.654	0.002
Step 2 PANSS disorganized duration of Sch	0.108	0.214	2.253	0.003

Independent variables entered in the analysis were: age, age at onset, illness duration, education, BMI, height, BPRS, and PANSS subscales.

We looked for a relationship between OCS and the age of onset of schizophrenia as well as its duration. Our data showed the existence of a statistical relationship between the presence of OCS at the onset of the illness and the duration of the schizophrenia process (Table 5).

**Table 5:** Effect of symptoms on scores of OCS in patients with schizophrenia.

	OCD scale	Onset of the Sch	Duration of Sch
OCD scale			
Pearson correlation	1	−0.220*	0.253**
Sig. (2-tailed)		0.024	0.009
Onset of Sch			
Pearson Correlation	−0.220*	1	−0.311**
Sig. (2-tailed)	0.024		0.001
Duration of Sch			
Pearson Correlation	0.253**	−0.311**	1
Sig. (2-tailed)	0.009	0.001	

## 4. Discussion

In our study, we find a female predominance. The explanation for this fact is related to the inclusion and exclusion criteria used. According the inclusion and exclusion criteria listed, most of the exclusion criteria cover the characteristics of male persons: abuse of psychoactive substances, alcohol, traumatic brain injuries, and so on. It is important to note and differences in compliance to treatment. It is easier to be accomplish in females then in males (74).

On the one hand, we find a higher percentage of patients with clinically pronounced ACS in the treatment-resistant group. On the other hand, we also found statistically significant higher values of obsessive–compulsive symptoms in the therapy-resistant group. These results of ours show that in the resistance group, patients have moderate OC symptoms, and in those in remission, OC symptoms are in the mild range of the scale. With this observation of ours, we confirm the data of some authors that the presence of symptoms from the domain of obsessive- compulsive phenomena is associated with a worse prognosis and a more severe course of the schizophrenic process (41–43, 75). These results do not support other authors’ observations about the lack of relationship between the expressiveness of OC symptoms and resistance in schizophrenia (44). We found a relationship between the severity of OCS and the duration of schizophrenia as well as its onset. These results of ours can be considered, on the one hand, as part of the general presentation of resistant schizophrenia, which is characterized by an earlier onset as well as a longer duration of symptoms (76). Our observation of a relationship between the duration of schizophrenia and the expression of OCS may be explained by the studies of other authors who indicated that “fixity and bizarreness of beliefs in OCD occur on a continuum from ‘none’ to ‘delusional intensity’ and may fluctuate within subjects” (77). The high values of the DOCS scale can also be considered as a result of the progression of the disease over time in the context of dementia praecox (78). The progression of the schizophrenic process over time can be explained by the changes in the energy in the neuron related to the mitochondrial DNA studies showing its involvement in various mental disorders both in patients with schizophrenia and patients with OCD (79–81).

This observation raises two questions: firstly, whether the appearance of OCS is related to resistance as part of it in the context of the observations of some authors that these patients need higher doses of antipsychotic drugs in general or secondly develop as a consequence of the progression of the disease in the course of time as part of the clinical picture and its dynamics. This is also the reason why other authors found fluctuating OCS in some patients with schizophrenia, up to 15.6% of patients with OCS and in 7% – development of OCS in patients who have not previously reported such (34).

The relationship with the disorganized symptoms that we found confirms the data of other authors on the correlation between the expressiveness of OCS and the disorganized symptoms on the PANSS scale for schizophrenia. We confirmed these observations. Furthermore, symptoms of disorganization have been identified as risk factors for a worse course of the schizophrenic process (82–84). Some evidence suggests that disorganization might be a stronger predictor of social functioning than reality distortion, presented with positive symptoms (85). Disorganization symptoms are directly related to negative and functional recovery (86). These data correspond to our observations of a statistically significant relationship of obsessive–compulsive symptoms with disorganized and partly with the negative ones and their higher values in patients with resistant schizophrenia. Our data on the relationship between obsessive–compulsive symptoms and the duration of the schizophrenia process confirm the data of other authors who found such a relationship (31).

How can we look for the likely explanation of these connections? Оn the one hand, schizophrenia and OCD share common pathogenetic mechanisms, frontostriatal deficits; on the other hand, in part of the patients with schizophrenia, progression in a neurodegenerative aspect is observed, which raises the question of involvement of the neuronal structures related to obsessive–compulsive symptoms in this process of progression (87–89). These data give reason to think about the development of the symptoms of OCD in the course of the development of the schizophrenic process. In a certain part of patients with schizophrenia, progression in neurodegenerative aspect was registered, on the other hand, it was found that the presence of disorganized symptoms was associated with a worse prognosis (90). The data related to the possible progression of the schizophrenic process provide an explanation of the fact why more pronounced obsessive–compulsive symptoms are observed in patients with resistance, why they are associated with more pronounced disorganized symptoms when assessed with the PANSS scale, as well as with the longer duration of the schizophrenia process (90, 91). The progression of the schizophrenic process is associated with a loss of neuronal populations both in patients with schizophrenia and in other diseases that are considered to be purely neurodegenerative (92, 93).

The relationship between obsessive–compulsive symptoms and the intake of antipsychotic medications is a controversial issue, because, on the one hand, some authors find a link between the intake of antipsychotic medications and the appearance of obsessive–compulsive symptoms (45, 46); on the other hand, antipsychotic medications are also used in OCD therapy, especially in those with an unsatisfactory response (53, 54). Other authors did not find a relationship between the intake of antipsychotics and obsessive–compulsive symptoms (47). From our research, we cannot draw a conclusion in support of one or the other thesis. What we found was that patients with resistance used much more antipsychotic medication compared to those in clinical remission, which is also inferred from the resistance of the symptomatology. On the other hand, patients with resistant schizophrenia have higher values in all subdomains of the PANSS scale. Bearing in mind that both OCD and psychotic symptoms are considered thought disorders, we can assume that the manifestation of psychotic symptoms (delusions and hallucinations) largely masks the appearance of OCD. In these cases, when using antipsychotic medications with an effect on psychotic symptoms, obsessive–compulsive symptoms that were present in the clinical picture but were not of clinical priority may appear.

We can conclude that in patients with resistant schizophrenia, there is a convergence of disorders involving the frontal lobe (20–22) with combined disorders in the thought process, behavior, which also reflect the degree of neuroprogression. In them, the mechanisms of neuroprogression and neurodegeneration exceed the possibilities of neuroregeneration and neuroprotection, as well as the potential of antipsychotic medications to restore the balance of these two parallel processes. With this dynamic development at the level of neurons, neuronal populations and connections in the CNS over time, we can expect a gradient deterioration in the absence of clinically relevant therapeutic attempts to stop neurodegenerative and stimulate neuroplasticity and neuroprotection processes.

In this regard, we can point out that some non-pharmacological methods of influence, such as TMS, in our opinion, are not sufficiently used in practice. TMS has shown its potential for therapeutic influence both in patients with schizophrenia and in those with obsessive–compulsive disorder (94–96).

### 4.1. Limitations

One of the limitations of our study is that we do not have information about the previous values of OCS to be able to make an observation over time, as well as to evaluate the influence of one or another factor on these symptoms. Another limitation of our study is related to the fact that we cannot make a connection between a particular antipsychotic medication and the presence of obsessive–compulsive symptoms. The patients observed by us have had previous psychotic episodes and have taken various medications, with a large number of them using more than one antipsychotic medication in search of an optimal therapeutic effect and minimal side effects. In the evaluation of patients, we have adhered to the consensus on resistance, in which equating drug therapy to chlorpromazine equivalents is required (although clinical practice has shown different effects of different medications in individual patient).

We have used the accepted criteria for resistance in patients with schizophrenia and we hope to follow the condition of the patients over time by being able to go along with both the stability of the criteria used and the dynamics of the points in the scales used.

## 5. Conclusion

We found a relationship between resistance in patients with schizophrenia and the severity of obsessive–compulsive symptoms. Positive symptoms are the ones that usually attract more attention from therapists, while other discrete behavioral changes often go unnoticed. Our results provide grounds in clinical practice to pay particular attention to the scale for disorganized symptoms as well as to the duration of the schizophrenic process in the assessment of patients. In the presence of expressiveness of the points on the disorganized scale or in the case of a longer duration of schizophrenia, it is appropriate to think about underlying obsessive–compulsive symptoms for which the corresponding therapeutic intervention is recommended. These observations of ours show that one of the recommendations in therapeutic interventions for resistant patients should also be related to the search for strategies to reduce obsessive–compulsive symptoms in the direction of remission as a clinical goal.

## Data availability statement

The raw data supporting the conclusions of this article will be made available by the authors, without undue reservation.

## Ethics statement

The studies involving human participants were reviewed and approved by Ethical Committee of University Hospital “Prof. Dr. Stoyan Kirkovich” Stara Zagora, protocol code TR3-02-242/30 December 2021. The patients/participants provided their written informed consent to participate in this study.

## Author contributions

Conceptualization, data collection and analysis by GP, writing, editing and graphics by PP. All authors listed have made a substantial, direct, and intellectual contribution to the work and approved it for publication.

## Conflict of interest

The authors declare that the research was conducted in the absence of any commercial or financial relationships that could be construed as a potential conflict of interest.

## Publisher’s note

All claims expressed in this article are solely those of the authors and do not necessarily represent those of their affiliated organizations, or those of the publisher, the editors and the reviewers. Any product that may be evaluated in this article, or claim that may be made by its manufacturer, is not guaranteed or endorsed by the publisher.
